# Stratifying cardiovascular benefits from GLP-1RA: a multisource analysis of patient-level CVOT and real-world data using AI-driven methods

**DOI:** 10.1186/s12933-025-02952-w

**Published:** 2025-10-17

**Authors:** Mario Luca Morieri, Enrico Longato, Veronica Sciannameo, Emily Donatiello, Paola Berchialla, Angelo Avogaro, Agostino Consoli, Gian Paolo Fadini

**Affiliations:** 1 Department of Medicine, University of Padova, Via Giustiniani 2, Padova, 35128 Italy; 2 Department of Information Engineering, University of Padova, Padova, Italy; 3 Centre for Biostatistics, Epidemiology and Public Health, Department of Clinical and Biological Sciences, University of Turin, Regione Gonzole 10, Orbassano, Italy; 4 Novo Nordisk Italia, SpA, Roma, Italy; 5 Department of Medicine and Aging Sciences, “G. d’Annunzio” University of Chieti- Pescara, Chieti, Italy; 6 Veneto Institute of Molecular Medicine, Padova, Italy

**Keywords:** Precision medicine, Cardiovascular prevention, GLP-1RA, Randomized clinical trial, Real-world evidence

## Abstract

**Background:**

It remains unclear whether certain individuals with type 2 diabetes (T2D) derive greater cardiovascular benefit from GLP-1 receptor agonists (GLP-1RAs). Here, we integrate individual-level data from cardiovascular outcome trials (CVOTs) and electronic health records (EHRs), applying machine learning methods to confirm the cardiovascular benefits of GLP-1RAs in real-world populations and to identify subgroups with enhanced treatment response.

**Methods:**

Data from two CVOTs (LEADER and SUSTAIN-6) and a large real-world study (DARWIN-T2D) were analyzed. We first transposed the hazard ratio (HR) for 3-point major adverse cardiovascular event (3P-MACE) from CVOTs to the real-world population. Then, we used PRISM (Patient Response Identifiers for Stratified Medicine) against 3P-MACE reduction by GLP-1RA in a training/test setting. Findings were validated with external cohorts of new-users of GLP-1RA or comparators (DPP-4 inhibitors or basal insulin).

**Results:**

Despite notable differences in clinical characteristics between CVOT and real-world patients, the real-world-transposed HRs for 3P-MACE closely paralleled those from CVOTs. PRISM identified subgroups with differential treatment responses, based on history of myocardial infarction (MI) or stroke and age. Participants aged over 71 years without MI/stroke (41% of the real-world population) had the greatest relative benefit (HR 0.46; 95% CI 0.24–0.89 in the test set) and a greater absolute risk reduction (ARR 4.5%, 95% CI 1.2–7.7) than other subgroups (Gail-Simon *p* = 0.02). The external validation cohort confirmed these results (HR 0.67; 95% CI 0.51–0.89 and ARR 3.8%, 95% CI 1.5–6.1) showing significant differences in absolute risk reduction (*p* < 0.05).

**Conclusions:**

This study supports the integration of individual data from CVOT with those from EHR to confirm the transposition of results from CVOT to real-world populations, and enables the identification and validation of subgroups with greater cardiovascular benefits from cardioprotective treatment such as GLP-1RA treatment. This precision medicine approach represents a new framework for deploying cardiovascular prevention strategies in T2D.

**Graphical abstract:**

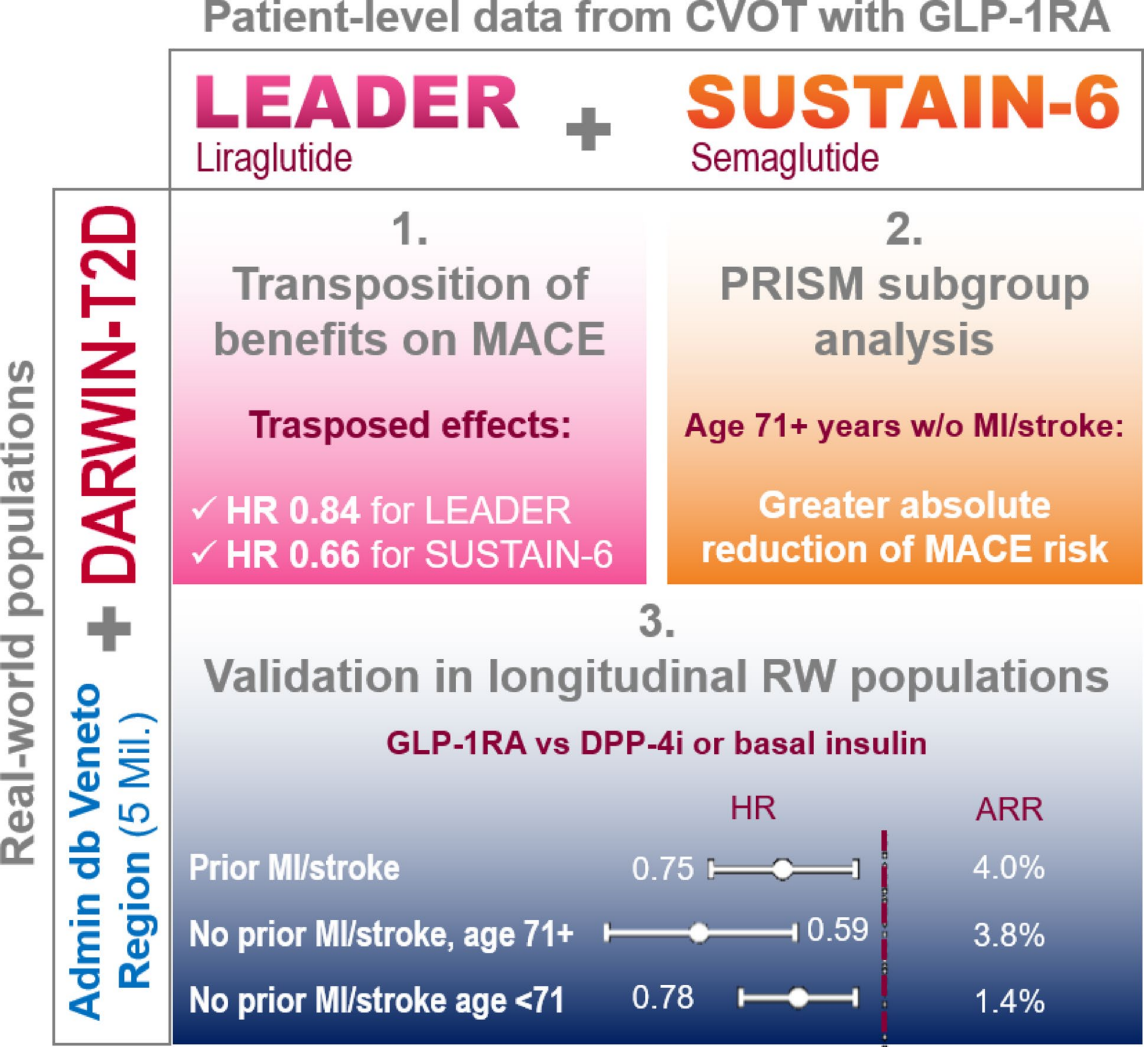

**Supplementary Information:**

The online version contains supplementary material available at 10.1186/s12933-025-02952-w.

## Research insight

**What is currently known about this topic?**

- GLP-1RAs reduce CV events in high-risk patients with type 2 diabetes.
- CVOT participants often differ from real-world patients and existing extrapolations used only aggregate trial data.
- It is unclear whether integrating individual-level data from CVOTs and EHRs can confirm GLP-1RA benefits in real-world patients in all subjects with type 2 diabetes and identify subgroups with greater cardiovascular benefit.

**What is the key research question?**

- Can trial-based cardiovascular outcomes of GLP-1RAs be transposed to real-world care populations, and are there identifiable subgroups with greater benefit?

**What is new?**

- GLP-1RA benefits were consistent between trial and real-world settings. Notably, elderly patients without prior cardiovascular events experienced an absolute CV risk reduction comparable to those on secondary prevention.

**How might this study influence clinical practice?**

- Identifies older adults in primary prevention with CV benefits like those in secondary prevention.
- Supports combining CVOT and real-world data to guide broader, risk-based GLP-1RA use in type 2 diabetes and highlight the value of precision medicine approaches in optimizing cardiovascular prevention.

## Background

Type 2 diabetes (T2D) accelerates atherosclerosis and the prevention of cardiovascular events remains a challenge in this population. However, not all individuals with T2D experience the same cardiovascular risk and not all are expected to draw similar benefits from the same treatment(s) [1].

In cardiovascular outcome trials (CVOTs), GLP-1RA effectively reduced the incidence of atherosclerotic cardiovascular events in people with T2D and prior cardiovascular disease or high risk for [2]. To what extent such results are generalizable to entire population of people with T2D is unclear, as transposing CVOT results to the real-world population of candidate patients can be challenging. Indeed, the trial setting is much different from routine clinical care, especially regarding patient characteristics [3]. Therefore, confirming transferability of trial findings to the real world is remarkably important for clinical practice [4]. We have previously shown that, using aggregate data from CVOT and individual data from the target real-world T2D population, it is possible to estimate (transpose) similar efficacy of GLP-1RA to a broader real-world population [5]. Taking into account the stratified estimates for several patient characteristics, the real world-transposed HR for MACE in the LEADER and SUSTAIN-6 trials remained very similar to those observed in the trial population. However, the approach based on aggregate data ignores the interdependency among clinical features and limits the identification of subgroups with different response to treatment. Here, we applied models that account for the joint distributions of multiple variables [6], overcoming such limitations using patient-level data and allowing for a more precise estimate of the treatment effect transposed to the real-world population. This enables to screen the real-world population for patient profiles that predict better responses to treatment in terms of cardiovascular event reduction.

Our goal was to test whether the cardiovascular protective effects of GLP-1RA observed in CVOTs can be effectively transposed to the real-world scenario. Then, we sought to identify subgroups of people with T2D from the real-world setting who are expected to benefit the most from cardiovascular protection exerted by GLP-1RA in the trials.

## Methods

### Study design

We collected individual patient-level data from the LEADER [7] and SUSTAIN-6 [8] CVOTs as well as from the DARWIN-T2D real world study (RWS) [9]. As summarized in Fig. S1A, after excluding patients with missing data for covariates in the RWS, we combined information from the two CVOTs with those of the RWS population (LEADER + DARWIN-T2D and SUSTAIN-6 + DARWIN-T2D). We modelled dependencies among variables in the combined populations and estimated appropriate weights for LEADER or SUSTAIN-6 participants. Parametric and non-parametric (i.e. Bayesian) models were used to estimate weights (as detailed in the Appendix). Such weights were then used to estimate the treatment effect of liraglutide or semaglutide transposed to the target population [4].

After evaluating the overall transferability of results from CVOTs to the target population, we assessed whether it was possible to identify subgroups of subjects in the RWS with greater benefit from treatment with GLP-1RA (Fig. S1B). To this end, we combined datasets from LEADER and SUSTAIN-6 and, after defining training and test sets, a machine learning approach was used to identify variables that modified the response to treatment in terms of the risk of MACE. These were validated on a test set and applied to the RWS to compute the proportions of individuals with T2D in the real-world population in each subgroup. Further external validation was performed in a longitudinal database previously used to test cardiovascular effectiveness of GLP-1RA [10, 11].

### Study populations

The LEADER (*n* = 9340) and SUSTAIN-6 (*n* = 3297) included patients with T2D aged 50 years or more and established CVD or aged 60 years or more and at least one additional cardiovascular risk factor [7]. After excluding participants with missing information on key variables of interest, our study included 8758 patients from the LEADER study and 3227 patients from SUSTAIN-6 study.

DARWIN-T2D was a retrospective multicenter study collecting data from 46 diabetes specialist outpatient clinics in Italy [12, 13]. The study recorded cross-sectional data on all patients with T2D aged 18 years or older at their last available visit, yielding to a population of ~ 281,000 patients, evaluated between 2015 and 2016. This is estimated to represent about 20% of the entire population of individuals with T2D attending diabetes clinics in Italy (note that only diabetes clinics and not GPs were authorized to prescribe GLP-1RA at that time [14]). Data on demographics, anthropometrics, risk factors, laboratory values, complications, and medications were collected. After exclusion of participants with missing information on key variables of interest, the present analysis included 72,736 individuals. The study was conducted according to the principles of the Declaration of Helsinki and approved by ethics committees at all participating centers. Patients’ informed consent was waived based on national regulations on retrospective studies with anonymous data.

### External validation cohort

We performed an external validation of the cardiovascular response to GLP-1RA in subgroups, using the administrative claims database of the Veneto Region [10, 11]. We employed two previous studies conducted by our group testing the cardiovascular effectiveness of GLP-1RA compared to DPP4i [10] or basal insulin [11]. These comparators are known to have neutral effects on MACE [15, 16]. As previously described, the Health Information Exchange system, covering a population of about 5 million inhabitants (330,193 with diabetes) was used to identify new users of GLP-1RA (exenatide, liraglutide, lixisenatide, dulaglutide), DPP-4i (sitagliptin, vildagliptin, alogliptin, linagliptin, saxagliptin) or basal insulins (detemir, glargine, degludec) from 2011 to 2018. To develop a pseudo-randomization approach, several clinical variables were used to obtain two propensity score matched (PSM) cohorts of new-users of GLP-1RA or active comparators.

### Outcomes

The primary outcome was the first occurrence of 3-point MACE (3P-MACE) as specified in the LEADER and SUSTAIN-6 study protocols (i.e. a composite outcome of non-fatal myocardial infarction or stroke and cardiovascular mortality). In the real-world longitudinal cohort, due to the lack of information on causes of death, we used a modified version of the 3P-MACE, including overall mortality instead of cardiovascular mortality, with cardiovascular mortality expected to cause 70% of overall mortality in people with T2D [17].

### Transposition analysis

To test the real-world validity of the treatment effect estimates obtained in the clinical trials, we applied weighted proportional hazards Cox regression analyses, using inverse odds of sampling weights as proposed by Westreich [18]. Further details are given in the Appendix.

#### Identification of subgroups with different treatment response

The training set was constructed by randomly selecting 70% of patients enrolled in LEADER and 70% of patients enrolled in SUSTAIN. The remaining 30% of LEADER and 30% of SUSTAIN participants were included in the test set.

To identify subgroups with different responses to treatment, we applied the statistical framework of the Patient Response Identifiers for Stratified Medicine (PRISM) tool, using the default ML algorithm for survival outcomes. Further details on the five steps of PRISM are given in the Appendix. Briefly the PRISM framework uses a multi-step ML approach: first, deriving a multivariable risk model for the outcome under control conditions; second, applying interaction modelling to detect key baseline modifiers of treatment benefit, selected through regularized regression (elastic net).

The overall clinical benefit of treatment in different groups was tested in the entire cohort of LEADER + SUSTAIN-6 with a Cox proportional hazard model including a covariate for trial identification. Sensitivity analyses were performed to account for possible imbalances of randomization in each subgroup (defined by a SMD >0.1 and p-value < 0.05). Predicted survival probability and number needed to treat (NNT) based on absolute risk reduction (NNT-ARR) were estimated according to Austin et al. [19, 20], at the time point closest to the median follow-up of RCTs (i.e. 3.6 years). Confidence intervals and standard errors for ARR were obtained with bootstrapping (1000 sampling with replacement). Quantitative absolute scale interactions were tested according to Gail and Simon methods [21].

Transferability and heterogeneity of treatment response were assessed using the following variables: age, sex, BMI, duration of diabetes, baseline HbA1c, history of CVD, history of HF, history of MI and stroke, hypertension, peripheral artery disease, eGFR and medications (metformin, sulphonylurea, thiazolidinediones, DPP4 inhibitors, RAS blockers, calcium channel blockers, beta blockers, diuretics, antiplatelet treatment, and statins). Urinary albumin/creatinine ratio (UACR) was used in the analyses specific to the LEADER study and not in SUSTAIN-6 due to high missingness (18%).

### External validation

The analyses were conducted following the same approach, combining the populations described in original studies [10, 11]. The balance between new-users of GLP-1RA and comparators (DPP-4i or basal insulin) was obtained with PSM, using the nearest neighbor method and the logit distance. The PS was estimated from the following variables: age, sex, claims-based history length, diabetes duration, presence of CV risk factors (dyslipidemia and hypertension), pre-existing vascular conditions (i.e. peripheral circulatory complications, myocardial infarction, ischemic heart disease, stroke or TIA, heart failure, cardiovascular disease), complications (i.e. neurological complications, ocular complications, renal complications, chronic kidney disease, severe hypoglycemia) and other conditions (cancer, chronic pulmonary disease, systemic inflammatory disease, Charlson comorbidity index), detailed information on glucose-lowering medications and other drugs. The PSM was originally built in two steps, GLP-1RA vs. DPP-4i and GLP-1RA vs. basal-insulin and it was possible for subjects to be included in the two studies (matching with replacement). The balance was assessed by evaluating the standardized mean difference (SMD) and good balance was defined as a SMD < 0.10. The association between GLP-1RA use and 3P-MACE was assessed with Cox regression models including an indicator for comparisons with DPP-4i or basal insulin. In subgroup analyses, the balance was verified and the main model was eventually adjusted for variables showing imbalance in the various strata. ARR and NNT were estimated as done for the CVOTs.

### Statistical analysis

Continuous variables are described as mean and standard deviations (SD). Categorical variables are presented as frequencies and percentages. Differences between groups for each variable were analyzed using t-test or chi-square for continuous or categorical variables, respectively. All records with at least one missing information were deleted from the analyses, as we applied a complete case scenario. The level of significance was set at 0.05, unless otherwise specified. All statistical analyses were performed using R version 4.2.1 and SAS v 9.4.

## Results

### Patient characteristics

The complete case datasets included 8758, 3227, and 72,736 participants from the LEADER, SUSTAIN-6 and DARWIN-T2D studies, respectively (Fig. S1). As expected, the clinical characteristics of patients included in the two trials were significantly different from those included in the real-world study (Table S1), with CVOT patients being younger and with higher HbA1c levels, greater prevalence of comorbidities, use of insulin and SU (*p* < 0.001for all).

### Results of trials can be transposed to the target real-world population

For transposition, we used two alternative approaches to estimate weights: Bayesian network (BN) and logistic regression (LR). The overall weight distribution estimated with BN and LR are shown in Fig. S2, while Fig. S3 shows the network built to estimate BN weights. Table 1 summarizes the results as reported in the original studies (all subjects), in the complete case datasets, and transposed results. Due to differences in clinical characteristics between the CVOTs and the target real-world population, the effective sample size used in the transposition analyses dropped significantly. However, in both trials, the transposition analyses revealed that the estimated effects of GLP-1RA on 3P-MACE in the DARWIN-T2D population were very similar to those reported in the CVOTs. Both estimates showed a trend towards larger effect in the transposed population. When formally tested, though, there was no significant interaction between weights and treatment in either study (Table S2).

**Table 1:** Results of transposition analyses. The table compares the HR for 3P-MACE obtained according to the approach used in the main analyses of LEADER and SUSTAIN-6 studies, or obtained after transposition with aggregate data or after transposition with patient-level data

	Leader		SUSTAIN-6	
	HR (95% CI)	Effective sample size	HR (95% CI)	Effective sample size
Main CVOT results				
Overall population	0.87 (0.78–0.97)	9340	0.74 (0.58–0.95)	3297
Complete case population	0.87 (0.78–0.97)	8758	0.72 (0.56–0.92)	3227
Transposed results				
Aggregate data	0.88 (0.77–0.99)		0.73 (0.47–0.99)	
Individual data				
Logistic-regression estimated weights	0.83 (0.69–0.99)	2048	0.55 (0.36–0.84)	688
Bayesian Network estimated weights	0.84 (0.74–0.96)	3934	0.66 (0.51–0.87)	1837

### Identification of subgroups with different treatment responses

**Fig. 1:**
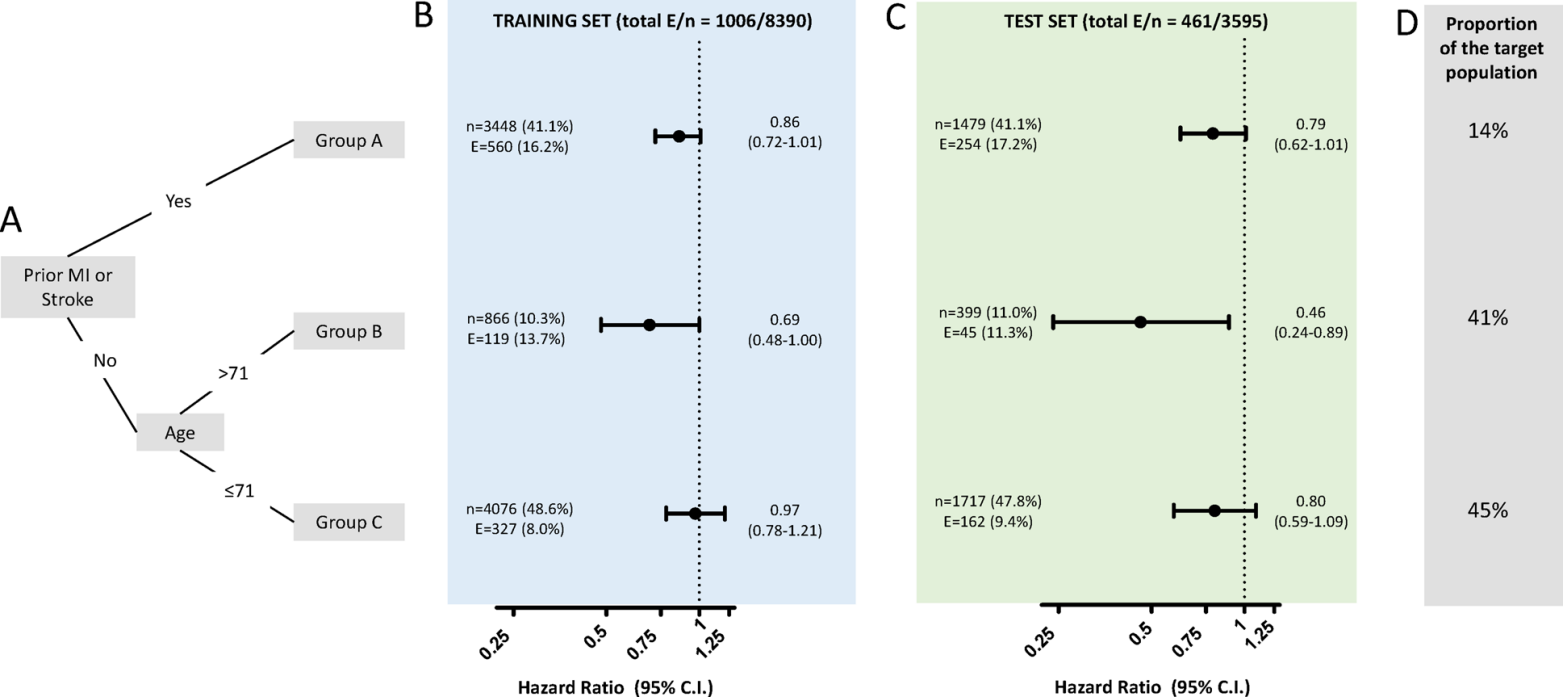
Heterogeneity of treatment response to GLP-1RA in CVOTs. **A** Classification tree from machine learning analysis to derive the three groups showing different response to GLP-1RA. **B**, **C** HR for 3P-MACE in the training set (B) and in the test set (**C**) in the three subgroups. **D** Proportion of patients represented in the real world population for each subgroups

**Fig. 2:**
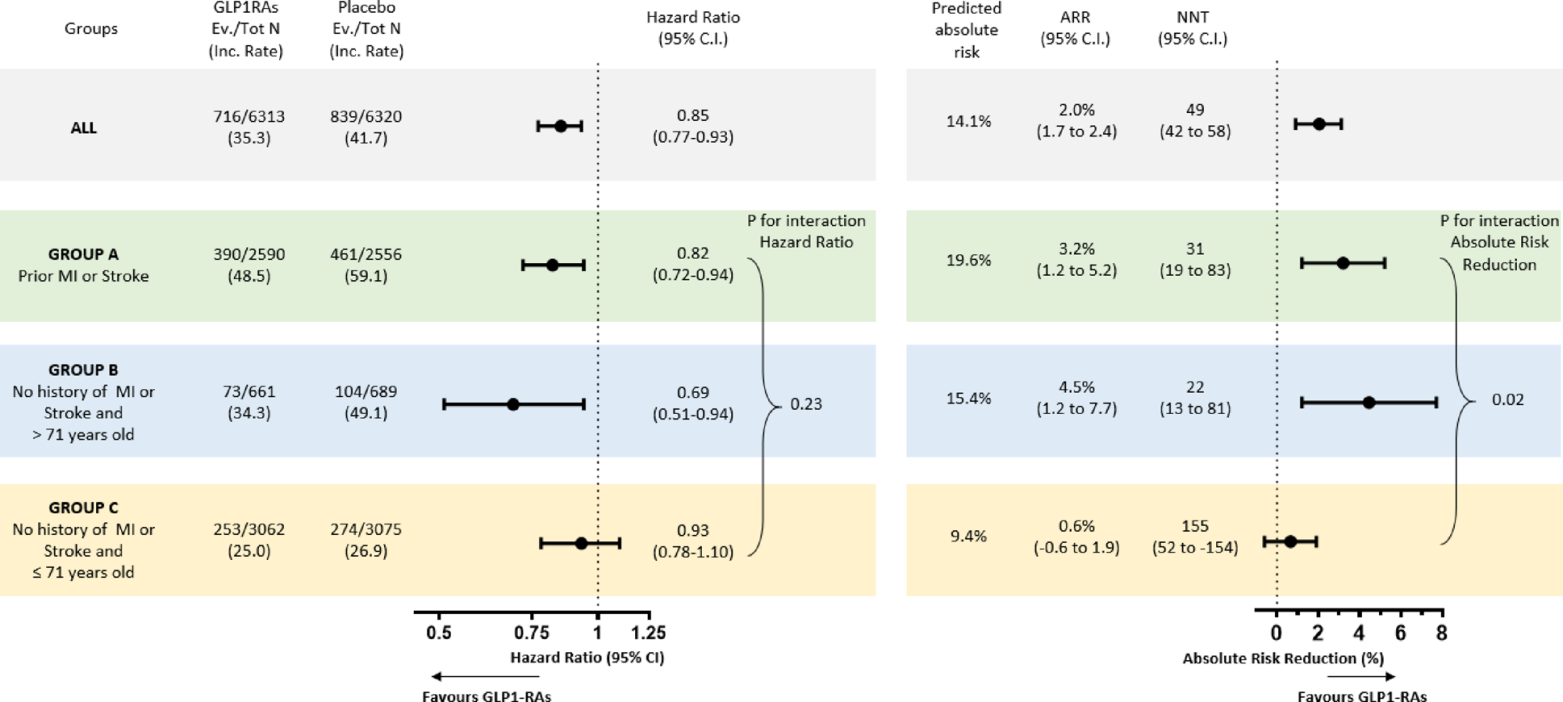
Clinical benefit of treatment with GLP-1RA vs. placebo in CVOTs. The predicted risk of MACE defined as “1 - predicted survival probability” was assessed with Cox proportional hazards regression models, including all covariates used for adjustments under the condition of all subjects being untreated, and assessed at median time of follow-up (i.e. 3.6 years). Absolute risk reduction (ARR) and number needed to treat (NNT) are estimated at the same time point. Interaction on the relative scale (HRs) was tested in the Cox regression, whereas interaction on the absolute scale (ARRs) was tested as described by Gail and Simon

After confirming that the results from CVOTs can be transposed, on average, to the real-world population, we explored whether there were subgroups of patients with greater benefit from treatment in CVOTs and in the target population. To do this, we combined data from the two CVOTs. With a data-driven machine-learning (ML) approach, we evaluated multiple variables as possible modulators of treatment response (the elastic net importance plot is depicted in Fig. S4). The two most important variables were (i) the presence/absence of a history of MI/stroke; (ii) age above/equal-below 71 years. The resulting algorithm (Fig. 1A) identified three subgroups with different cardiovascular response to GLP-1RA (Group A: patients with prior history of CVD; Group B: patients without a history of CVD and aged > 71 years; Group C: patients without a history of CVD and aged ≤ 71 years). Results obtained in the training set (Fig. 1B) were confirmed in the test set (Fig. 1C). Participants without a history of CVD and aged > 71 years (“group B”) showed a greater relative benefit (training set HR 0.69, 95% C.I. 0.48-1.00; test set HR 0.46, 95% C.I. 0.24–0.89), as compared to the other two groups (with CVD or younger than 71 years). Remarkably, group B represented only 10.6% of the entire RCT cohorts, but it accounted for 41.0% of the target real-world population of the DARWIN-T2D study (Fig. 1D). To increase statistical power to estimate the differences in clinical benefit of GLP-1RA treatment across the three subgroups, we combined the training and test sets of the two CVOTs (Fig. 2). Notably, while the differences in the rates of 3P-MACE on the relative scale were not statistically significant (interaction *p* > 0.05), there was a significant interaction (Gail-Simon *p* for quantitative interaction = 0.02) for the absolute risk reduction (or NNT). Indeed, the trend towards greater benefit in the relative scale in group B was combined with the highest absolute risk in this group, especially when compared to group C (9.6% vs. 5.4%), leading to an absolute greater clinical benefit among subjects of group B. Remarkably, there was no cross-over interaction, and the differences between groups were driven by larger benefits rather than by the identification of groups where GLP-1RA was not effective or detrimental.

Several sensitivity analyses were performed. The balance of randomization was tested within each subgroup (Table S3) and adjustments for variables showing imbalance with SMD > 0.1 (diabetes duration, prevalence of HF, and statin use) did not influence the overall results (Table S4). Moreover, since the clinical characteristics of patients identified by these two indicators (prior MI/stroke and age) in the RCTs were significantly different from those in the real-world setting (Table S5), we also performed weighted analyses. The transposed HR from CVOT to real-world population stratified by these modulators (as in transposition analyses shown above) confirmed the main results (Table S6).

### External validation

**Fig. 3:**
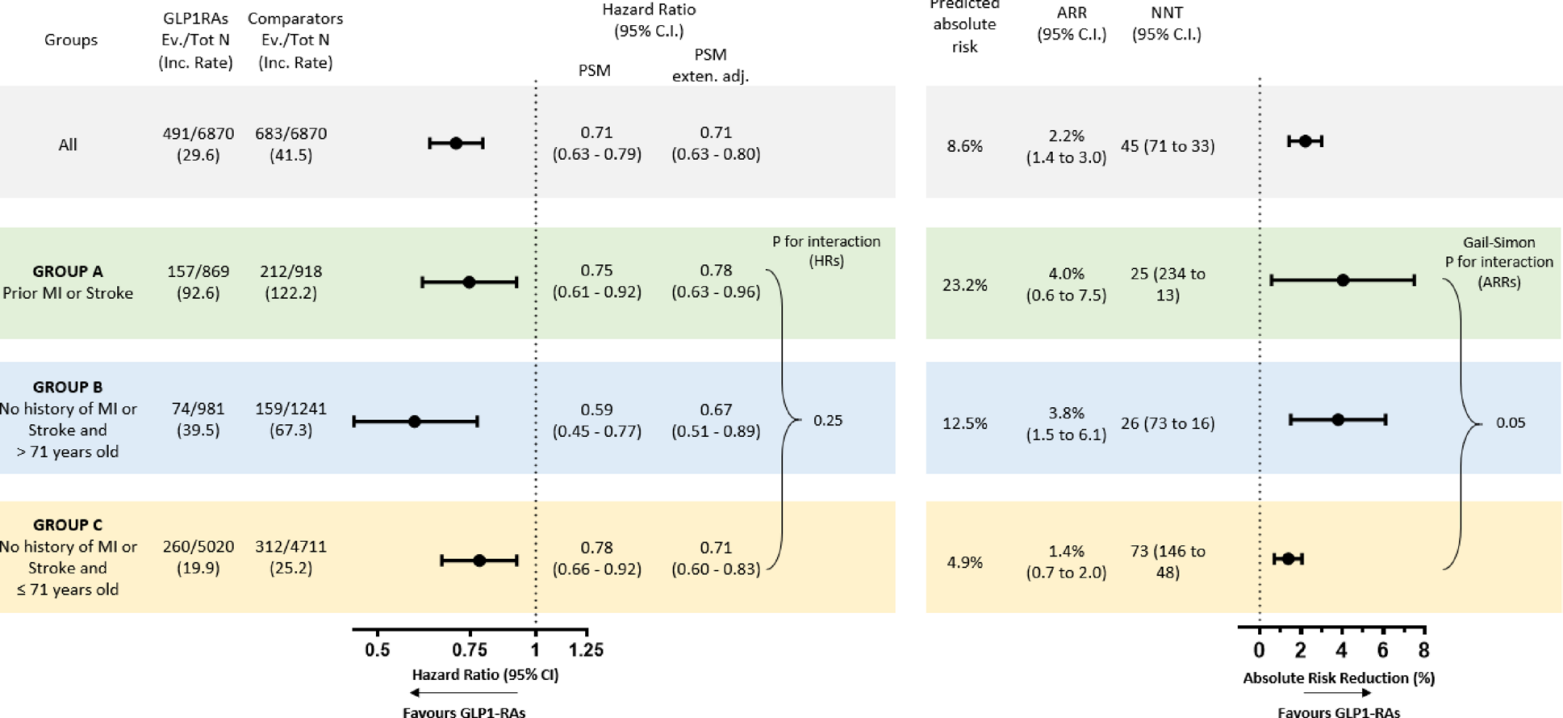
External validation of the clinical benefit of treatment with GLP-1RA versus DPP4i or basal insulin. Absolute risk reduction (ARR) and number needed to treat (NNT) are estimated at the same time point. Interaction on the relative scale (HRs) was tested in the Cox regression, whereas interaction on the absolute scale (ARRs) was tested as described by Gail and Simon

We tested our findings in an external longitudinal real-world population. In the absence of a placebo arm, to simulate the exposure observed in trials, we compared new-users of GLP-1RA with new users of diabetes medications with an established neutral effect on 3P-MACE (namely DPP-4i and basal insulin). The populations included 6708 propensity-score matched subjects per group initiating GLP-1RA or comparators. The two populations were well balanced (Table S7). After a median follow-up of 25 months (IQR 12–43) 1,174 subjects experienced 3P-MACE. In the overall cohort, use of GLP-1RA was associated with a significant reduction of 3P-MACE rate as compared to those treated with comparators (HR 0.71; 95% C.I. 0.63 to 0.79; *p* < 0.0001). When we analyzed the three subgroups identified above (Fig. 3), the reduction in the rates of 3P-MACE was significant in all subgroups, with group B showing the numerically greatest benefit (HR 0.59; 95% C.I. 0.45–0.77; *p* = 0.0002). The differences between groups were blunted after adjustments for residual imbalances within subgroups (Table S7) and there was no significant interaction on the relative scale (interaction *p* > 0.05). However, as observed in the CVOTs, when the clinical benefit was assessed on the absolute scale, which accounts for the differences in baseline risk across groups, there was a significant Gail-Simon quantitative interaction (*p* < 0.05), with subjects in group B experiencing a net clinical benefit (ARR 3.8%) similar to those observed among subjects with prior history of stroke or myocardial infarction (ARR 4.0%), and greater than in group C (ARR 1.4%; *p* = 0.03) (Fig. 3).

## Discussion

This study provides three significant and novel insights by integrating individual-level data from two landmark CVOTs and two real-world cohorts. First, we show that the cardiovascular benefits of GLP-1RA observed in CVOTs are transferrable to the real-world setting, even after accounting for the differences between trial participants and patients under routine care. Second, an extensive research into heterogeneity of the response to GLP-1RA revealed no subgroup deriving no benefit from treatment. This highlights the broad applicability of GLP-1RA across different patient phenotypes even under routine care. Third, it was possible to identify patients with a higher baseline risk, in whom GLP-1RA exerted a trend greater reduction in the relative risk of MACE and a significantly greater absolute risk reduction. Specifically, individuals aged 71 or older in primary cardiovascular prevention (i.e. without a history of MI or stroke) showed a significantly higher clinical benefit comparable to patients in secondary CVD prevention. This is particularly important given the aging of the population and the progressive decline in the rates of cardiovascular disease in the population with and without diabetes [22, 23]. It should be noted that the average age of people with T2D in Italy approximates 70 years and only 15% have a history of prior cardiovascular events [24], which highlights how distant is the population of patients seen under routine care from that of patients enrolled in CVOTs. Indeed, the group of patients showing the best cardiovascular response to GLP-1RA was underrepresented in CVOTs but represented a remarkable proportion (41%) of the real-world population. This identifies a clear opportunity for more widespread treatment and cardiovascular prevention.

The strengths of our study lie in the use of individual-level data from CVOTs and RWS, which enhances the reliability and applicability of the findings across diverse settings. This approach allowed us to address complex relationships in clinical characteristics and interdependencies among variables, advancing beyond previous aggregate data analyses [5]. More importantly, a recent international consensus on precision medicine issued a call for action on using individual data from large CVOT to gain more insight on their general results [25, 26]. While individual-data analyses confirmed that findings of CVOTs can be transferred to the real-world population “on average”, the availability of detailed data is the ideal condition to search for subgroups of individuals with different responses to treatment. Our data-driven approach showed that GLP-1RA were effective across various subgroups without significant cross-over interactions, but with significant quantitative interactions (i.e. subgroups of subjects with greater cardiovascular benefit). Such differences were more evident when observed as absolute rates, which is more relevant than relative risks (i.e. HR) from a clinical and cost-effectiveness perspective. Indeed, precision medicine approaches can identify groups with a larger relative effect or groups with higher baseline risk, where the absolute benefit is also bigger. In this case, we identified a combination of the two, with a trend for lower HR that, combined with a higher baseline risk, projected a significant clinical benefit on the absolute scale of event rates. Remarkably, the same interaction was consistently replicated in an external real-world cohort, with a trend towards interaction on the relative scale that was enhanced by differences in baseline risk across subgroups, yielding a significant difference in absolute risk reduction.

Current treatment guidelines/consensus statements recommend using cardioprotective agents (SGLT2i and GLP-1RA) independently from glucose control for patients with high cardiovascular risk, prioritizing GLP-1RA in the presence of atherosclerotic disease [27]. Some suggest the use of these agents as first-line treatment for T2D in patients with overt CVD, such as prior MI or stroke [28]. Our findings offer a new perspective, showing that patients older than 71 years without overt CVD derive a clinical benefit from treatment with GLP-1RA that is similar or even greater than do patients with overt CVD. For patients aged ≥ 71 years in primary prevention versus those in secondary prevention, respectively, the NNT was 31 versus 22 in CVOTs (over 3.6 years) and 25 versus 26 in the real world (over 2.1 years). In simple terms, once started on a GLP-1RA, elderly individuals without overt CVD are expected to gain the same absolute benefit as do patients with overt CVD. This might be particularly relevant from the perspective of patients and healthcare systems, both being more sensitive to absolute risk than relative risk reduction. More so, if we consider that the subgroup of elderly patients in primary prevention represents a much larger proportion of the real-world T2D population. The aging of the population and the decline in overall mortality among T2D patients highlight the clinical relevance of our findings.

By suggesting that elderly patients with T2D would benefit from treatment with GLP-1RA, we are not denying the importance of intervening earlier in the natural course of the disease. Even a smaller benefit obtained over a relatively short treatment (2–4 years) in the younger population is expected to translate into a greater benefit on the lifetime risk of CVD. Indeed, patients with T2D onset in their 40s and 50s have the worst diabetes-related cardiovascular morbidity and mortality [17]. Support for GLP-1RA efficacy in younger populations with lower baseline CVD risk also comes from the GRADE trial. In this large randomized study, participants had a mean age of 57 years, relatively short diabetes duration and predominantly free of established CVD. In post hoc analyses, those randomized to liraglutide experienced a lower rate of MACE events compared with those assigned to other glucose-lowering agents (glimepiride, insulin glargine, or sitagliptin) [29]. In these terms, our study confirms the broad benefits of GLP-1RA against cardiovascular events also in the younger population under routine care.

We wish to acknowledge some study limitations. First, it may be argued that, in the absence of a significant interaction for HR, there is no manifest difference of the GLP-1RA effect across groups. Yet, results similar to ours were reported in other settings and deemed to be highly clinically relevant. For example, re-analyses of the effects of PCSK9 inhibitors stratified by LDL-cholesterol levels [30, 31], and of the effect of the combination aspirin/rivaroxaban stratified by diabetes status [32] showed significant differences in the ARR across groups even with similar HR.

Second, to identify subjects with greater benefit from treatment, we used a data-driven approach instead of testing modulators known for their biological plausibility. Our unbiased approach has intrinsic limitations, i.e. prior MI/stroke and age might not be true modulators of the response to GLP-1RA but rather identify a subgroup of individuals with other characteristics that, in turn, drive the different response. The same features, however, may select patients with varying characteristics in different settings. Moreover, while data-driven approaches may identify specific thresholds for continuous variables (e.g., 71 years of age in our population), these should be interpreted cautiously and considering the overall clinical context rather than a rigid threshold. Our external validation of the main finding supports the validity of the ML-derived classification but, further studies focused on biology-driven modulators (e.g. circulating GLP-1 levels or genetic variants on GLP1/GLP1R loci) are of interest to deliver complementary information. At the same time, it should be acknowledged that, despite our efforts to combine multiple CVOTs with individual-level data, the ability to detect more refined subgroups remains limited. Prior MI/stroke and age were selected by our machine learning algorithm as the most relevant variables, based on their top-ranking importance in the elastic net model (figure S4). However, other variables—such as HbA1c, concomitant statin use, and sex—also ranked highly and may contribute to further stratification. Future studies, ideally leveraging larger pooled CVOT datasets, may help uncover additional treatment effect modifiers and enable more granular subgroup identification. We also recognize that we identified a subgroup deriving greater benefit from treatment despite being underrepresented in CVOTs, thus limiting the reliability of the classification.

Third, some variables were unavailable in the real-world database (e.g. ethnicity), and we could not incorporate them into the models. While multiple ethnicities are likely represented, the majority of participants are expected to be of European ancestry. Moreover, most of individuals were on metformin at baseline, thus our results are mainly generalizable to patients receiving GLP-1RA therapy in combination with metformin. In addition, there may be some heterogeneity between RWS and CVOTs in the definitions of variables and outcomes. It should also be noted that comparator choice (active versus placebo) and the definition of 3P-MACE in the external replication cohort may generate different HR in the real-world versus the CVOTs. Notably, we wish to underline that we only focused on cardiovascular events, disregarding that GLP-1RA can provide substantial benefits on glycemic and body weight control, other risk factors, liver disease, as well as kidney disease.

Finally, though we used two large real-world databases for transferability and external validation, they were limited to the Italian population. Given that all real-world patients were followed under diabetology specialist care, extrapolation to other settings (e.g. general practitioners) and to populations with very different T2D phenotypes (e.g. Asian / Pacific) needs caution. Remarkably it has been recently shown how subjects from Asia could have a larger benefit versus white population [33], further research using diverse international datasets is therefore recommended. Nonetheless, we believe that the proof-of-concept framework of our study may well apply to other geographical areas using suitable real-world data.

## Conclusion

In summary, our study confirms that the cardiovascular efficacy estimates observed in CVOTs for GLP-1RA are transposable (and therefore generalizable) to the real-world setting, on average. At the same time, while we found no group of subjects in real life experiencing no benefit from GLP-1RA treatment, it is possible to identify new subgroups of individuals with unexpectedly good response, extending a strong indication for GLP-1RA use to elderly patients in primary cardiovascular prevention.

## Supplementary Information

Below is the link to the electronic supplementary material.

Supplementary Material 1.

## Data Availability

The data that support the findings of this study are available on request from the corresponding author, but restrictions apply due to compliance with privacy regulations.
