# Supplementary material for: Stratifying cardiovascular benefits from GLP-1RA: a multisource analysis of patient-level CVOT and real-world data using AI-driven methods
Source: Cardiovasc Diabetol. 2025 Oct 17;24:401. doi: 10.1186/s12933-025-02952-w (PMC12535053; doi:10.1186/s12933-025-02952-w)
Supplement: Supplementary file 1 — Supplementary Material 1. [file 12933_2025_2952_MOESM1_ESM.docx]

**Appendix**

**Supplemental method section**

**Transposition analysis**

Let S indicates the indicator variable stating if a subject *i* is in the trial (S_i_ = 1) or if the subject *i* is in the target population (S_i_ = 0). Then, let **X**_i_ = (X_1_, X_2_, …, X_n_), i=1,…,N be the vector of baseline characteristics. The weights w_i_ were computed for each subject *i* as expressed in equation (1):

*
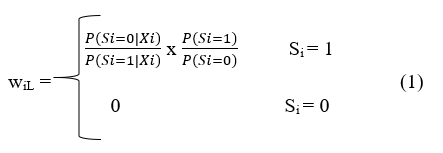
*

If an individual *i* did not participate in the clinical trial, his/her weight was 0. If she/he did participate, her/his weight is the inverse of their conditional probability of being sampled divided by their conditional probability of not being sampled, based on their baseline characteristics **X**_i_, multiplied by the ratio of their unconditional probability of being sampled to their unconditional probability of not being sampled. Such inverse odds weights are appropriate when the study sample is not a subset of the target population and we refer to this situation as a problem of “transportability” rather than “generalizability” (1). Weights smaller than 0.01 and greater than 3 were trimmed respectively to 0.01 and 3.

We estimated the probabilities in equation (1) via two different approaches: the first, is the more traditional parametric approach, i.e., the logistic regression. More in detail, a generalized linear model with binomial family and logit link function was used. The second approach, is a Machine Learning (ML) non-parametric approach, i.e., the Bayesian Network (BN) which computes the quantities of interest as conditional probabilities. The Peter-Clark stable algorithm with a 100-fold bootstrap was applied for the construction of the BN to address the sample variability, to learn conditional dependencies between the variables(2). Then, a more robust BN was obtained by averaging 25 BNs, taking into account only the conditional dependencies between variables which appeared in at least 95% of times and using a Bayesian posterior estimator with a uniform prior matching that in the Bayesian Dirichlet equivalent score (3,4). Predictions are performed with the “bayes-lw” method. All the available baseline variables **X**_i_ are used for both models.

Then, a weighted univariate proportional hazards Cox regression analysis was performed to obtain Hazard Ratio (HR) estimates and their 95% confidence intervals (95% CIs). For the weighted Cox regression models, Kish's Effective Sample Sizes (ESS) are computed as follows:


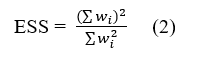


**Application of the PRISM (Patient Response Identifiers for Stratified Medicine) tool**

First, PRISM reduces the set of covariates with a variable selection algorithm (Elastic Net (5)). Second, Patient Level Estimates (PLE) of the treatment effect are computed for each subject in the trial in the counterfactual setting (i.e., the expected difference in response if the patient had received both treatments), using random forest algorithm (“ranger” R package (6)). For survival, the output is the restricted mean survival time treatment difference. Third, subgroups of patients with similar treatment effect are identified through a regression tree (lmtree with log-rank score transformation on outcome Y, then model based partitioning (MOB) Ordinary Least Squares (OLS) is fitted through “partykit” R package (7,8)). Finally, subgroup specific parameters estimation is performed through proportional hazards Cox regression model and Kaplan Meier curves. The algorithm trained to identify subgroups was then validated on a test set. The identified subgroups were then applied on DARWIN-T2D to compute the proportions of patients of the real world who were classified in the subgroups.

**Supplemental tables**

**Table S1. Baseline characteristics of patients in LEADER, SUSTAIN-6 and DARWIN-T2D (RWS)**. Continuous variables are expressed as mean (SD) and categorical value as n (%). All comparison showed significant differences between RCTs and targeted RW population (with the exception of diabetes duration and eGFR, that where similar between in the RWS and SUSTAIN-6 RCT, with p>0.05).

|  | SUSTAIN-6 | | LEADER | |
| --- | --- | --- | --- | --- |
|  | RWS | RCT | RWS | RCT |
| n | 72736 | 3227 | 68025 | 8758 |
| Male | 42295 (58.1) | 1963 (60.8) | 39435 (58.0) | 5623 (64.2) |
| Age, years | 70.52 (10.16) | 64.70 (7.36) | 70.52 (10.11) | 64.25 (7.18) |
| Diabetes duration, years | 14.01 (9.16) | 13.93 (8.15) | 14.00 (9.13) | 12.91 (7.92) |
| HbA1c, % | 7.27 (1.16) | 8.68 (1.45) | 7.24 (1.14) | 8.68 (1.51) |
| BMI, kg/m^2^ | 29.33 (5.33) | 32.85 (6.19) | 29.31 (5.31) | 32.58 (6.26) |
| eGFR, ml/min/1.73m^2^ | 74.9 (23.2) | 75.6 (22.8) | 74.6 (22.7) | 79.1 (22.0) |
| Hypertension, % | 61503 (84.6) | 3131 (97.0) | 57558 (84.6) | 8491 (97.0) |
| Prior MI, % | 8716 (12.0) | 1056 (32.7) | 8110 (11.9) | 2709 (30.9) |
| Prior MI or stroke, % | 10207 (14.0) | 1435 (44.5) | 9488 (13.9) | 3492 (39.9) |
| Prior heart failure, % | 1545 (2.1) | 560 (17.4) | 1422 (2.1) | 1229 (14.0) |
| Prior PAD, % | 6223 (8.6) | 49 (1.5) | 5821 (8.6) | 1074 (12.3) |
| Antidiabetic treatments |  |  |  |  |
| Metformin, % | 46597 (64.1) | 2366 (73.3) | 43669 (64.2) | 6970 (79.6) |
| Sulphonylurea, % | 18455 (25.4) | 1387 (43.0) | 16892 (24.8) | 4621 (52.8) |
| Thiazolidinediones, % | 3408 (4.7) | 76 (2.4) | 3242 ( 4.8) | 561 (6.4) |
| DPP4 inhibitors, % | 15568 (21.4) | 5 (0.2) | 14412 (21.2) | 6 (0.1) |
| Insulin, % | 24196 (33.3) | 1879 (58.2) | 22597 (33.2) | 4086 (46.7) |
| Other treatments |  |  |  |  |
| Anti-hypertensive, % | 56356 (77.5) | 3076 (95.3) | 52809 (77.6) | 8289 (94.6) |
| ACEi/ARBs, % | 47076 (64.7) | 1110 (34.4) | 44143 (64.9) | 2855 (32.6) |
| Calcium-channel blockers, % | 18392 (25.3) | 1053 (32.6) | 17257 (25.4) | 2908 (33.2) |
| Beta blockers, % | 22624 (31.1) | 1888 (58.5) | 21169 (31.1) | 4973 (56.8) |
| Diuretics, % | 13808 (19.0) | 1257 (39.0) | 12883 (18.9) | 3760 (42.9) |
| Antiplatelet agents, % | 35114 (48.3) | 2102 (65.1) | 33021 (48.5) | 5649 (64.5) |
| Statins, % | 43691 (60.1) | 2394 (74.2) | 41137 (60.5) | 6483 (74.0) |

**Table S2. Interaction between treatments and weights in SUSTAIN-6 and LEADER cohorts.**

|  | **SUSTAIN-6** | | | | | |  |
| --- | --- | --- | --- | --- | --- | --- | --- |
|  | Logistic Regression | | | Bayesian Network | | |  |
| Predictors | Estimate | SE | p | Estimate | SE | p |  |
| Treatment * weights | -0.034 | 0.079 | 0.67 | -0.038 | 0.070 | 0.59 |  |
|  | **LEADER** | | | | | | |
|  | Logistic Regression | | | Bayesian Network | | | |
| Predictors | Estimate | SE | p | Estimate | SE | p | |
| Treatment * weights | -0.193 | 0.183 | 0.29 | -0.130 | 0.171 | 0.45 | |

**Table S3: Balance of randomization in combined CVOT population (LEADER and SUSTAIN-6) stratified by subgroups with different cardiovascular response to GLP-1RA.** Continuous variables are expressed as mean (SD) and categorical value as n (%). Imbalances were assessed with standardized mean difference and defined by SMD≥0.10.

|  | **All (SUSTAIN-6 + LEADER)** | | | **GROUP A** | | | **GROUP B** | | | **GROUP C** | | |
| --- | --- | --- | --- | --- | --- | --- | --- | --- | --- | --- | --- | --- |
|  | **Placebo** | **GLP1-RA** | **SMD** | **Placebo** | **GLP1-RA** | **SMD** | **Placebo** | **GLP1-RA** | **SMD** | **Placebo** | **GLP1-RA** | **SMD** |
| **n** | 6320 | 6313 |  | 2556 | 2590 |  | 689 | 661 |  | 3075 | 3062 |  |
| **Male** | 64.4 ± 7.3 | 64.3 ± 7.2 | 0.02 | 63.6 ± 7.6 | 63.5 ± 7.5 | 0.02 | 75.6 ± 3.5 | 75.6 ± 3.3 | 0.00 | 62.6 ± 5.2 | 62.6 ± 5.1 | 0.01 |
| **Age (years)** | 3980 (63.0) | 4023 (63.7) | -0.02 | 1813 (70.9) | 1854 (71.6) | -0.01 | 399 (57.9%) | 381 (57.6%) | 0.01 | 1768 (57.5%) | 1788 (58.4%) | -0.02 |
| **Diabetes duration (years)** | 13.0 ± 8.1 | 13.1 ± 8.0 | -0.01 | 12.9 ± 8.2 | 12.8 ± 8.1 | 0.01 | 15.4 ± 9.1 | 16.8 ± 9.5 | -0.15 | 12.7 ± 7.6 | 12.6 ± 7.4 | 0.01 |
| **HbA1c (%)** | 8.7 ± 1.5 | 8.7 ± 1.5 | -0.04 | 8.7 ± 1.5 | 8.8 ± 1.6 | -0.06 | 8.3 ± 1.3 | 8.4 ± 1.3 | -0.10 | 8.7 ± 1.5 | 8.7 ± 1.5 | -0.01 |
| **BMI (kg/m^2^)** | 32.6 ± 6.2 | 32.6 ± 6.3 | -0.01 | 32.2 ± 6.1 | 32.2 ± 5.9 | 0.00 | 31.5 ± 5.5 | 31.6 ± 5.8 | -0.02 | 33.1 ± 6.5 | 33.2 ± 6.6 | -0.01 |
| **eGFR (ml/min/1.73 m^2^)** | 135.7 ± 17.5 | 135.9 ± 17.7 | -0.01 | 134.9 ± 17.6 | 134.6 ± 17.7 | 0.01 | 137.5 ± 17.6 | 138.1 ± 18.6 | -0.03 | 136.0 ± 17.3 | 136.5 ± 17.4 | -0.03 |
| **Systolic blood Pressure (mmHg)** | 77.0 ± 10.1 | 77.2 ± 10.2 | -0.01 | 76.8 ± 10.2 | 77.1 ± 10.1 | -0.03 | 74.6 ± 10.1 | 74.1 ± 10.7 | 0.05 | 77.7 ± 9.9 | 77.9 ± 10.2 | -0.02 |
| **Diastolic Blood Pressure (mmHg)** | 78.4 ± 22.2 | 78.1 ± 22.4 | 0.01 | 79.7 ± 21.9 | 79.6 ± 22.2 | 0.01 | 64.5 ± 19.8 | 63.7 ± 20.2 | 0.04 | 80.4 ± 21.9 | 80.0 ± 22.0 | 0.02 |
| **Hypertension, n (%)** | 6035 (95.5) | 6063 (96.0) | -0.03 | 2459 (96.3) | 2506 (96.8) | -0.03 | 656 (95.2%) | 639 (96.7%) | -0.07 | 2920 (95.0%) | 2918 (95.3%) | -0.02 |
| **Prior MI, n (%)** | 1941 (30.7) | 1992 (31.6) | -0.02 | 1941 (75.9) | 1992 (76.9) | -0.02 | 0 (0.0%) | 0 (0.0%) | 0.00 | 0 (0.0%) | 0 (0.0%) | 0.00 |
| **Prior MI or Stroke, n (%)** | 2556 (40.4) | 2590 (41.0) | -0.01 | 2556 (100) | 2590 (100) |  | 0 (0.0%) | 0 (0.0%) | 0.00 | 0 (0.0%) | 0 (0.0%) | 0.00 |
| **Prior Heart Failure, n (%)** | 940 (14.9) | 937 (14.8) | 0.00 | 406 (15.9) | 414 (16.0) | 0.00 | 105 (15.2%) | 77 (11.6%) | 0.11 | 429 (14.0%) | 446 (14.6%) | -0.02 |
| **Prior PAD, n (%)** | 625 (9.9) | 600 (9.5) | 0.01 | 205 (8.0) | 199 (7.7) | 0.01 | 89 (12.9%) | 87 (13.2%) | -0.01 | 331 (10.8%) | 314 (10.3%) | 0.02 |
| **Antidiabetic treatments, n (%)** | 6125 (96.9) | 6096 (96.6) | 0.02 | 2473 (96.8) | 2487 (96.0) | 0.04 | 661 (95.9%) | 632 (95.6%) | 0.02 | 2991 (97.3%) | 2977 (97.2%) | 0.00 |
| **Metformin, n (%)** | 3088 (50.2) | 2993 (48.9) | 0.03 | 1275 (51.2) | 1255 (50.1) | 0.02 | 337 (50.6%) | 326 (51.4%) | -0.02 | 1476 (49.2%) | 1412 (47.4%) | 0.04 |
| **Sulphonylurea, n (%)** | 4806 (78.1) | 4751 (77.6) | 0.01 | 1939 (77.9) | 1953 (78.0) | 0.00 | 453 (68.0%) | 412 (65.0%) | 0.06 | 2414 (80.5%) | 2386 (80.0%) | 0.01 |
| **Thiazolidinediones, n (%)** | 3074 (50.0) | 3066 (50.1) | 0.00 | 1211 (48.7) | 1210 (48.3) | 0.01 | 345 (51.8%) | 316 (49.8%) | 0.04 | 1518 (50.6%) | 1540 (51.6%) | -0.02 |
| **Insulin, n (%)** | 320 (5.2) | 331 (5.4) | -0.01 | 108 (4.3) | 105 (4.2) | 0.01 | 35 (5.3%) | 39 (6.2%) | -0.04 | 177 (5.9%) | 187 (6.3%) | -0.02 |
| **Other treatment** |  |  |  |  |  |  |  |  |  |  |  |  |
| **Anti-hypertensive, n (%)** | 5830 (94.3) | 5879 (95.2) | -0.04 | 2398 (94.7) | 2440 (95.6) | -0.04 | 628 (93.9%) | 619 (95.7%) | -0.08 | 2804 (94.1%) | 2820 (94.9%) | -0.03 |
| **ACEi/ARBs, n (%)** | 2049 (33.1) | 2033 (32.9) | 0.00 | 779 (30.8) | 741 (29.0) | 0.04 | 247 (36.9%) | 265 (41.0%) | -0.08 | 1023 (34.3%) | 1027 (34.5%) | 0.00 |
| **Calcium-channel Blockers, n (%)** | 2015 (32.6) | 2056 (33.3) | -0.02 | 800 (31.6) | 793 (31.1) | 0.01 | 238 (35.6%) | 226 (34.9%) | 0.01 | 977 (32.8%) | 1037 (34.9%) | -0.04 |
| **Beta Blockers, n (%)** | 3488 (56.4) | 3585 (58.1) | -0.03 | 1726 (68.1) | 1770 (69.3) | -0.03 | 337 (50.4%) | 323 (49.9%) | 0.01 | 1425 (47.8%) | 1492 (50.2%) | -0.05 |
| **Diuretics, n (%)** | 2588 (41.9) | 2575 (41.7) | 0.00 | 1039 (41.0) | 1032 (40.4) | 0.01 | 340 (50.8%) | 309 (47.8%) | 0.06 | 1209 (40.6%) | 1234 (41.5%) | -0.02 |
| **Antiplatelet agents, n (%)** | 3956 (64.0) | 4025 (65.2) | -0.03 | 1854 (73.2) | 1896 (74.3) | -0.02 | 396 (59.2%) | 398 (61.5%) | -0.05 | 1706 (57.2%) | 1731 (58.2%) | -0.02 |
| **Statins, n (%)** | 4536 (73.4) | 4601 (74.5) | -0.03 | 2013 (79.5) | 2095 (82.1) | -0.07 | 436 (65.2%) | 452 (69.9%) | -0.10 | 2087 (70.0%) | 2054 (69.1%) | 0.02 |

**Table S4:** Association between GLP1-RAs treatments and MACE by subgroup after adjustments for variables showing imbalances despite randomization (duration of diabetes, heart failure, statin treatment, HbA1c).

| **Groups** | **HR (95% CI)** | **HR (95% CI)**  **with further adjustments** |
| --- | --- | --- |
| A | 0.82 (0.72-0.94) | 0.82 (0.71-0.94) |
| B | 0.69 (0.51-0.94) | 0.69 (0.51-0.94) |
| C | 0.93 (0.78-1.10) | 0.90 (0.76-1.08) |

**Table S5.** Comparison of best responders (group B) in the targeted real-world population and RCTs (LEADER and SUSTAIN-6 combined).

|  | **DARWIN** | **CVOTs** | **p** |
| --- | --- | --- | --- |
| n | 29770 | 866 |  |
| Male | 15123 (50.8) | 496 (57.3) | <0.001 |
| Age (years) | 16.13 (9.76) | 16.15 (9.32) | 0.959 |
| Diabetes duration (years) | 78.60 (4.89) | 75.64 (3.39) | <0.001 |
| HbA1c - % | 7.25 (1.09) | 8.36 (1.27) | <0.001 |
| BMI - kg/m^2^ | 28.50 (4.98) | 31.64 (5.57) | <0.001 |
| Hypertension | 25871 (86.9) | 838 (96.8) | <0.001 |
| Prior PAD | 2924 (9.8) | 104 (12.0) | 0.039 |
| Prior Heart Failure | 931 (3.1) | 119 (13.7) | <0.001 |
| Antidiabetic treatments | 26938 (90.5) | 861 (99.4) | <0.001 |
| Insulin | 10430 (35.0) | 438 (50.6) | <0.001 |
| Metformin | 16423 (55.2) | 576 (66.5) | <0.001 |
| Sulphonylurea | 8721 (29.3) | 451 (52.1) | <0.001 |
| Thiazolidinediones | 987 (3.3) | 51 (5.9) | <0.001 |
| DPP4 inhibitors | 6127 (20.6) | 0 (0.0) | <0.001 |
| Anti-Hypertensive treatment. | 24049 (80.8) | 817 (94.3) | <0.001 |
| ACEi/ARBs | 19809 (66.5) | 331 (38.2) | <0.001 |
| Ca-Channel Blockers | 8432 (28.3) | 305 (35.2) | <0.001 |
| Beta Blockers | 8865 (29.8) | 441 (50.9) | <0.001 |
| Diuretics | 7154 (24.0) | 430 (49.7) | <0.001 |
| Anti-platelet trt. | 14834 (49.8) | 532 (61.4) | <0.001 |
| Statins | 17105 (57.5) | 594 (68.6) | <0.001 |

**Table S5.** Crude and real world-transposed HR for MACE risk reduction with GLP-1RA in LEADER and SUSTAIN-6 studies (complete case combined cohorts), stratified by subgroups.

Analyses adjusted by trials, estimates are reported as treated vs placebo as HR (95% CI). ESS, Effective Sample Size.

|  | **Non-Weighted** | | **Weighted** | | | |
| --- | --- | --- | --- | --- | --- | --- |
|  |  |  | **Weights based on Logistic Regression** | | **Weights based on Bayesian Network** | |
|  | **Estimate** | **Sample Size** | **Estimate** | **ESS** | **Estimate** | **ESS** |
| **Group A** | 0.82 (0.71-0.94) | 4897 | 0.86 (0.68-1.08) | 1082 | 0.83 (0.71-0.97) | 2358 |
| **Group B** | 0.69 (0.51-0.93) | 1294 | 0.36 (0.22-0.61) | 304 | 0.61 (0.43-0.87) | 629 |
| **Group C** | 0.92 (0.77-1.09) | 5792 | 0.85 (0.65-1.13) | 1350 | 0.80 (0.65-0.98) | 2781 |

**Table S7: Balance of propensity scored matched subjects treated with GLP1-RA or control treatments (DPP-4i or basal insulin) in real world setting.**

Continuous variables are expressed as mean (SD) and categorical value as n (%). Imbalances were assessed with standardized mean difference and defined by SMD≥0.10.

|  | **ALL SUBJECTS** | | | **GROUP A** | | | **GROUP B** | | | **GROUP C** | | |
| --- | --- | --- | --- | --- | --- | --- | --- | --- | --- | --- | --- | --- |
|  | **Control** | **GLP1-RAs** | **SMD** | **Control** | **GLP1-RAs** | **SMD** | **Control** | **GLP1-RAs** | **SMD** | **Control** | **GLP1-RAs** | **SMD** |
| **n** | 6870 | 6870 |  | 918 | 869 |  | 1241 | 981 |  | 4711 | 5020 |  |
| **Age (years)** | 63.2 ± 10.8 | 63.3 ± 8.9 | -0.01 | 67.3 ± 9.5 | 66.4 ± 7.9 | 0.10 | 77.3 ± 4.6 | 75.6 ± 3.6 | 0.42 | 58.7 ± 8.4 | 60.4 ± 7.4 | -0.21 |
| **Sex (male)** | 2708 (39.4) | 2713 (39.5) | 0.00 | 192 (20.9) | 183 (21.1) | 0.00 | 712 (57.4) | 502 (51.2) | 0.12 | 1804 (38.3) | 2028 (40.4) | -0.04 |
| **History length (years)** | 53 (36-68) | 54 (35-68) | 0.00 | 58 (42-71) | 60 (42-71) | 0.00 | 62 (47-73) | 62 (47-74) | 0.00 | 50 (32-65) | 52 (32-65) | -0.03 |
| **Diabetes duration (years)** | 97 (56-149) | 98 (55-148) | 0.01 | 108 (61-162) | 109 (64-164) | -0.05 | 124 (72-173) | 128 (75-172) | -0.02 | 89 (51-139) | 90 (50-137) | 0.00 |
| **Hypertension, n (%)** | 5666 (82.5) | 5727 (83.4) | -0.02 | 902 (98.3) | 856 (98.5) | -0.02 | 1160 (93.5) | 896 (91.3) | 0.08 | 3604 (76.5) | 3975 (79.2) | -0.06 |
| **Dyslipidemia, n (%)** | 4819 (70.1) | 4828 (70.3) | 0.00 | 851 (92.7) | 824 (94.8) | -0.09 | 929 (74.9) | 749 (76.4) | -0.03 | 3039 (64.5) | 3255 (64.8) | -0.01 |
| **CVD, n (%)** | 1056 (15.4) | 976 (14.2) | 0.03 | 918 (100.0) | 869 (100.0) | 0.00 | 65 (5.2) | 35 (3.6) | 0.08 | 73 (1.5) | 72 (1.4) |  |
| **Myocardial infarction, n (%)** | 393 (5.7) | 379 (5.5) | 0.01 | 393 (42.8) | 379 (43.6) | -0.02 | 0 (0.0) | 0 (0.0) | 0.00 | 0 (0.0) | 0 (0.0) |  |
| **ischemic heart disease, n (%)** | 709 (10.3) | 698 (10.2) | 0.01 | 709 (77.2) | 698 (80.3) | -0.08 | 0 (0.0) | 0 (0.0) | 0.00 | 0 (0.0) | 0 (0.0) |  |
| **Stroke or TIA, n (%)** | 275 (4.0) | 224 (3.3) | 0.04 | 275 (30.0) | 224 (25.8) | 0.09 | 0 (0.0) | 0 (0.0) | 0.00 | 0 (0.0) | 0 (0.0) |  |
| **Heart failure, n (%)** | 192 (2.8) | 148 (2.2) | 0.04 | 88 (9.6) | 60 (6.9) | 0.10 | 54 (4.4) | 27 (2.8) | 0.09 | 50 (1.1) | 61 (1.2) | -0.01 |
| **PAD, n (%)** | 63 (0.9) | 47 (0.7) | 0.03 | 27 (2.9) | 28 (3.2) | -0.02 | 12 (1.0) | 8 (0.8) | 0.02 | 24 (0.5) | 11 (0.2) | 0.05 |
| **CKD, n (%)** | 121 (1.8) | 100 (1.5) | 0.02 | 36 (3.9) | 27 (3.1) | 0.04 | 22 (1.8) | 23 (2.3) | -0.04 | 63 (1.3) | 50 (1.0) | 0.03 |
| **COPD, n (%)** | 2076 (30.2) | 2120 (30.9) | -0.01 | 320 (34.9) | 289 (33.3) | 0.03 | 456 (36.7) | 342 (34.9) | 0.04 | 1300 (27.6) | 1489 (29.7) | -0.05 |
| **Systemic inflamm. dis, n (%)** | 156 (2.3) | 158 (2.3) | 0.00 | 20 (2.2) | 12 (1.4) | 0.06 | 31 (2.5) | 12 (1.2) | 0.09 | 105 (2.2) | 134 (2.7) | -0.03 |
| **Cancer, n (%)** | 731 (10.6) | 723 (10.5) | 0.00 | 125 (13.6) | 114 (13.1) | 0.01 | 198 (16.0) | 161 (16.4) | -0.01 | 408 (8.7) | 448 (8.9) | -0.01 |
| **Charlson index** | 0.40 ± 1.02 | 0.34 ± 0.95 | 0.06 | 1.08 ± 1.34 | 0.98 ± 1.33 | 0.08 | 0.44 ± 1.13 | 0.43 ± 1.23 | 0.01 | 0.25 ± 0.85 | 0.21 ± 0.73 | 0.05 |
| **PAD, n (%)** | 100 (1.5) | 78 (1.1) | 0.03 | 47 (5.1) | 46 (5.3) | -0.01 | 20 (1.6) | 15 (1.5) | 0.01 | 33 (0.7) | 17 (0.3) | 0.05 |
| **Insulin ever, n (%)** | 493 (7.2) | 488 (7.1) | 0.00 | 83 (9.0) | 69 (7.9) | 0.04 | 108 (8.7) | 71 (7.2) | 0.05 | 302 (6.4) | 348 (6.9) | -0.02 |
| **Metformin, n (%)** | 6207 (90.3) | 6189 (90.1) | 0.01 | 833 (90.7) | 775 (89.2) | 0.05 | 1097 (88.4) | 845 (86.1) |  | 4277 (90.8) | 4569 (91.0) | -0.01 |
| **Sulfonylureas, n (%)** | 3541 (51.5) | 3549 (51.7) | 0.00 | 459 (50.0) | 470 (54.1) | -0.08 | 762 (61.4) | 608 (62.0) | -0.01 | 2320 (49.2) | 2471 (49.2) | 0.00 |
| **SGLT2-i, n (%)** | 229 (3.3) | 257 (3.7) |  | 33 (3.6) | 53 (6.1) | -0.12 | 25 (2.0) | 36 (3.7) | -0.10 | 171 (3.6) | 168 (3.3) | 0.02 |
| **DPP-4i, n (%)** | 1650 (40.6) | 1652 (40.7) | 0.00 | 252 (46.3) | 226 (42.6) | 0.08 | 343 (45.7) | 280 (47.5) | -0.04 | 1055 (38.1) | 1146 (39.0) | -0.02 |
| **Pioglitazone, n (%)** | 1009 (14.7) | 1008 (14.7) | 0.00 | 90 (9.8) | 103 (11.9) | -0.07 | 191 (15.4) | 157 (16.0) | -0.02 | 728 (15.5) | 748 (14.9) | 0.02 |
| **ACE inhibitors, n (%)** | 4904 (71.4) | 4922 (71.6) | -0.01 | 770 (83.9) | 737 (84.8) | -0.03 | 1016 (81.9) | 765 (78.0) | 0.10 | 3118 (66.2) | 3420 (68.1) | -0.04 |
| **Diuretics, n (%)** | 1305 (19.0) | 1345 (19.6) | -0.01 | 360 (39.2) | 292 (33.6) | 0.12 | 371 (29.9) | 275 (28.0) | 0.04 | 574 (12.2) | 778 (15.5) | -0.10 |
| **Beta blockers, n (%)** | 2369 (34.5) | 2415 (35.2) | -0.01 | 656 (71.5) | 665 (76.5) | -0.12 | 475 (38.3) | 354 (36.1) | 0.05 | 1238 (26.3) | 1396 (27.8) | -0.03 |
| **Anti-hypertensive other, n (%)** | 560 (8.2) | 589 (8.6) | -0.02 | 93 (10.1) | 101 (11.6) | -0.05 | 145 (11.7) | 97 (9.9) | 0.06 | 322 (6.8) | 391 (7.8) | -0.04 |
| **Statins, n (%)** | 4210 (61.3) | 4222 (61.5) | 0.00 | 807 (87.9) | 770 (88.6) | -0.02 | 825 (66.5) | 678 (69.1) | -0.06 | 2578 (54.7) | 2774 (55.3) | -0.01 |
| **Fibrates or omega-3, n (%)** | 786 (11.4) | 783 (11.4) | 0.00 | 175 (19.1) | 183 (21.1) | -0.05 | 96 (7.7) | 81 (8.3) | -0.02 | 515 (10.9) | 519 (10.3) | 0.02 |
| **Ezetimibe, n (%)** | 177 (2.6) | 176 (2.6) | 0.00 | 55 (6.0) | 61 (7.0) | -0.04 | 25 (2.0) | 18 (1.8) | 0.01 | 97 (2.1) | 97 (1.9) | 0.01 |
| **Anti-platelet agents, n (%)** | 2351 (34.2) | 2365 (34.4) | 0.00 | 762 (83.0) | 737 (84.8) | -0.05 | 478 (38.5) | 377 (38.4) | 0.00 | 1111 (23.6) | 1251 (24.9) | -0.03 |

**Figure S1. Study design and flowchart**.

A) Transposition analysis. B) Best responder analysis.

**A)**


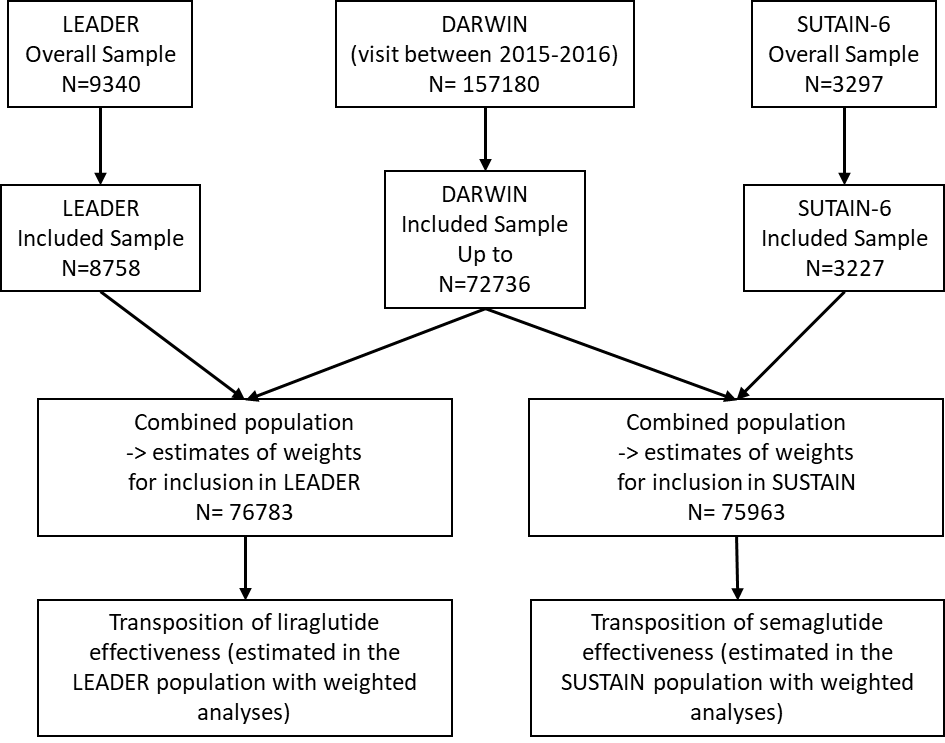


B)


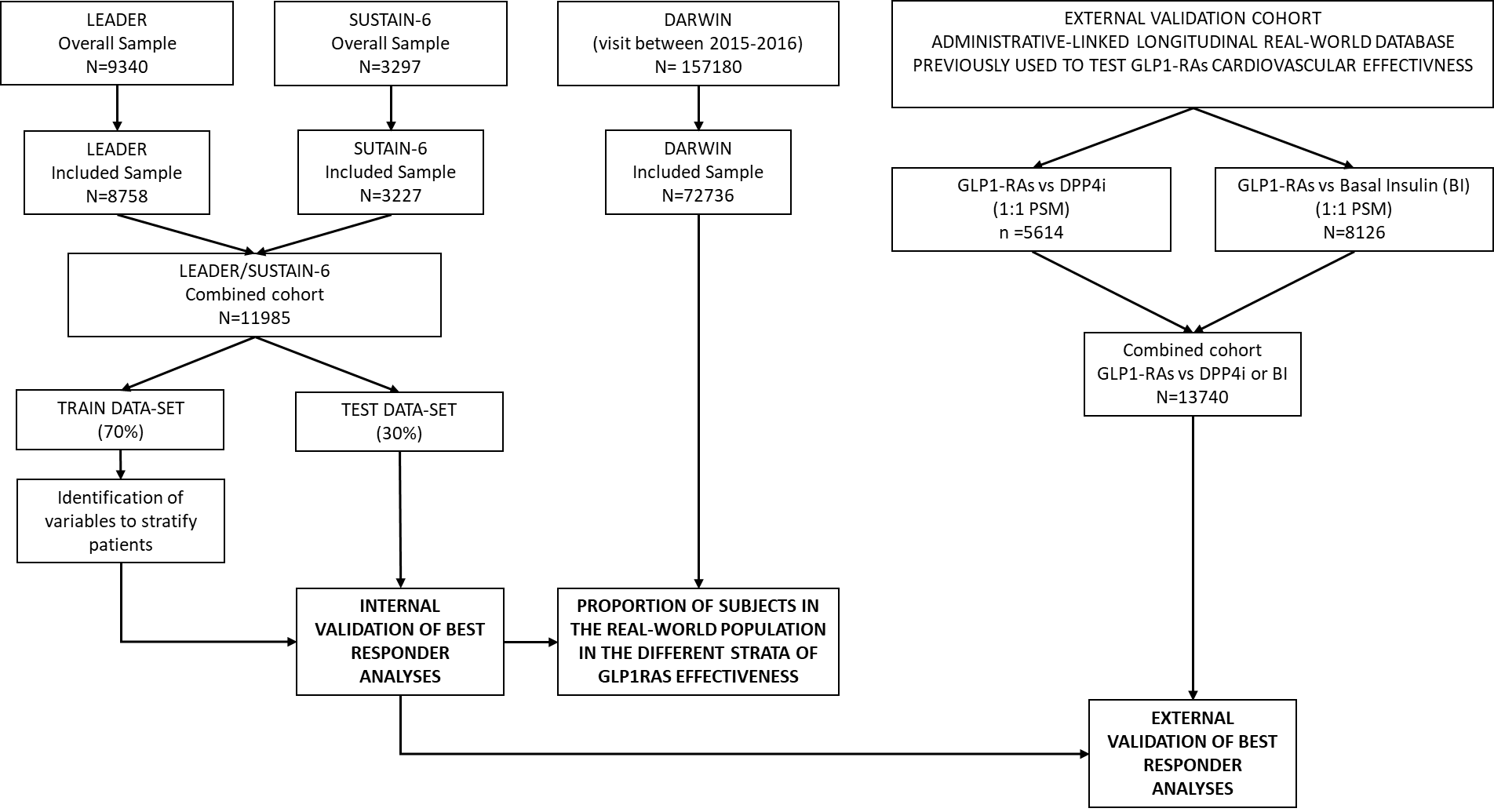


**Figure S2**. Distribution of weights estimated with Bayesian network (BN) and logistic regression (LR) in the SUSTAIN-6 and LEADER trials.

**Figure S3. Bayesian networks in the SUSTAIN-6 and LEADER trials**.

**Figure S4. Elastic net importance**

Supplementary materials references

1. Westreich D, Edwards JK, Lesko CR, Stuart E, Cole SR. Transportability of Trial Results Using Inverse Odds of Sampling Weights. Am J Epidemiol 2017;186:1010-1014.

2. M.H. CDM. Order-Independent Constraint-Based Causal Structure Learning. Journal of Machine Learning Research 2014;15:3921-3962.

3. Broom BM, Do KA, Subramanian D. Model averaging strategies for structure learning in Bayesian networks with limited data. BMC Bioinformatics 2012;13 Suppl 13:S10.

4. Azzimonti L. CG, Zaffalon M.,. Hierarchical estimation of parameters in Bayesian networks. Computational Statistics & Data Analysis 2019;137:67-91.

5. Zou H, Hastie T. Regularization and Variable Selection Via the Elastic Net. Journal of the Royal Statistical Society Series B: Statistical Methodology 2005;67:301-320.

6. Schwarz DF, Konig IR, Ziegler A. On safari to Random Jungle: a fast implementation of Random Forests for high-dimensional data. Bioinformatics 2010;26:1752-8.

7. Zeileis A, Hothorn T, Hornik K. Model-Based Recursive Partitioning. Journal of Computational and Graphical Statistics 2008;17:492-514.

8. Seibold H, Zeileis A, Hothorn T. Model-Based Recursive Partitioning for Subgroup Analyses. The international journal of biostatistics 2016;12:45-63.
